# Nanostructured Biomaterials for Tissue Engineered Bone Tissue Reconstruction

**DOI:** 10.3390/ijms13010737

**Published:** 2012-01-11

**Authors:** Gardin Chiara, Ferroni Letizia, Favero Lorenzo, Stellini Edoardo, Stomaci Diego, Sivolella Stefano, Bressan Eriberto, Zavan Barbara

**Affiliations:** 1 Department of Histology, Microbiology and Medical Biotechnology, University of Padova, Via G. Colombo 3, 35100 Padova, Italy; E-Mails: Gardinc@unipd.it (G.C.); Ferronil@unipd.it (F.L.); 2 Department of Periodontology, School of Dentistry, University of Padova, Via Venezia 90, 35100 Padova, Italy; E-Mails: Stomacid@unipd.it (S.D.); eribertobresan@unipd.it (B.E.)

**Keywords:** nanostructures, stem cells, bone, tissue engineering

## Abstract

Bone tissue engineering strategies are emerging as attractive alternatives to autografts and allografts in bone tissue reconstruction, in particular thanks to their association with nanotechnologies. Nanostructured biomaterials, indeed, mimic the extracellular matrix (ECM) of the natural bone, creating an artificial microenvironment that promotes cell adhesion, proliferation and differentiation. At the same time, the possibility to easily isolate mesenchymal stem cells (MSCs) from different adult tissues together with their multi-lineage differentiation potential makes them an interesting tool in the field of bone tissue engineering. This review gives an overview of the most promising nanostructured biomaterials, used alone or in combination with MSCs, which could in future be employed as bone substitutes. Recent works indicate that composite scaffolds made of ceramics/metals or ceramics/polymers are undoubtedly more effective than the single counterparts in terms of osteoconductivity, osteogenicity and osteoinductivity. A better understanding of the interactions between MSCs and nanostructured biomaterials will surely contribute to the progress of bone tissue engineering.

## 1. Introduction

Bone defects, which can arise from several causes such as trauma, tumors, infection or bone diseases (*i.e.*, osteoporosis), are very common in our society. Nowadays, most therapies for bone defects are based on autografts or allografts [1]. Autografts are tissues grafted into a new position in or on the body of the same individual. They are still considered the gold standard for bone transplantation because they possess all the three elements for new bone growth: osteoconductivity, osteogenicity, and osteoinductivity. Osteoconduction refers to the ability of the graft to ensure adhesion, survival, and proliferation of osteogenic cells, providing an interconnected structure through which new cells can migrate and new vessels can form. An osteogenic graft, on the other hand, implies the presence of osteoblasts at the fusion site that are able to synthesize new bone directly. Finally, osteoinduction refers to the ability of a graft to induce non-differentiated stem cells or osteoprogenitor cells from the surrounding tissue to differentiate into bone-forming osteoblasts [2]. The other key element for a successful bone replacement is osteointegration, which describes the surface bonding between the host bone and the grafting material [3]. The use of autologous bone grafts in surgical practice is common, and the iliac crest is the most frequently chosen donor site as it represents a good source in terms of quality and quantity of cancellous and cortical bone [4]. However, harvesting of iliac crest bone graft or autograft in general is associated with numerous drawbacks, including extra-surgery time, post-operative pain, hematoma formation, blood loss, nerve injury, infection and cosmetic defects, only to name a few [5,6]. Allografts, which are the transplantation of tissue -in this case bone tissue-between genetically nonidentical individuals of the same species, are an attractive alternative to autografts. Bone allografts may be cancellous, cortical, or a combination of each; and it is possible to manufacture customized types, such as dowels, strips, and chips [7]. Fresh allografts are rarely used as they might have the risk of disease transmission and immune response. For this reason, fresh-frozen or freeze-dried bone allografts are the preferred forms. Fresh-frozen allografts are more osteoinductive and have stronger mechanical properties but greater infection and rejection potential than freeze-dried allografts. Conversely, freeze-dried allografts do not produce immune host responses but are weaker and less osteoinductive. This is the consequence of the process of preparation, which implies allograft freezing, drying up to 5% of water, and sterilization with ethylene oxide or gamma irradiation. The shelf-life of freeze-dried allografts is indefinite, whereas fresh-frozen bone can be stored 1 year at −20 °C or 5 years at −70 °C [8]. Another form of allogenic bone is demineralized bone matrix (DBM), which is produced through decalcification of cortical bone and further processing, including chemical and radiation treatments. The result is a denatured form of the remaining protein matrix, which does not provide structural strength, but can serve as a biologic osteoconductive scaffold in a structurally stable environment. Though a variety of allografts exists with different biological properties, they all possess reduced osteoinductive properties and no cellular component, because donor grafts are devitalized via irradiation or freeze-drying processing [9].

Considering the complications associated with tissue-based graftings, bone-graft substitutes represent an efficient alternative to natural tissues in the treatment of bone defects. The ideal bone graft substitute should be osteoconductive, osteogenic, osteoinductive, biocompatible, biodegradable, structurally similar to bone, easy to use clinically and cost-effective [10]. A large number of bone-graft alternatives are commercially available, and they generally consist of scaffolds made of natural or synthetic biomaterials that promote the migration, proliferation and differentiation of cells for bone regeneration. Biomaterials mostly used as bone substitutes include collagen, ceramics made from calcium phosphate, such as hydroxyapatite (HA) and β-tricalcium phosphate (β-TCP), and glass ceramics [11]. Collagen, and in particular type I collagen, is the most abundant protein in the extracellular matrix (ECM) of bone. Thanks to its structure, collagen promotes mineral deposition, vascular ingrowth and noncollagenous matrix protein binding, providing a favorable environment to bone regeneration. Collagen functions poorly as a graft material, but if coupled with bone morphogenetic proteins (BMPs), osteoprogenitor precursors, or HA, it enhances significantly incorporation of grafts [12]. Ceramics are synthetic scaffolds that have shown to induce a biological response similar to that of bone. Both HA and β-TCP are highly biocompatible, with HA being more permanent in the implant site compared with TCP-ceramics. Alone, these synthetic ceramics do not possess osteogenic or osteoinductive properties; as a consequence, they are often modified or combined with autogenous bone in order to improve their functionality [13]. Glass ceramics are bioactive glass consisting of calcium, phosphorus, and silicon-dioxide, showing both the characteristics of osteointegration and osteoconduction. Glass ceramics have greater mechanical strength compared with calcium phosphate preparations, but at the same time they appear very brittle and subject to fracture [14].

From what has emerged so far, most of the current strategies for bone regeneration exhibit relatively satisfactory results, as the majority of the available biomaterials appear to be predominantly osteogenic or osetoinductive or, on the contrary, purely osteoconductive [15]. A promising approach that could overcome the limitations of current therapies for producing synthetic grafts relies on tissue engineering techniques. Bone tissue engineering techniques aim to combine the use of cells (for osteogenesis) seeded in three-dimensional (3D) biocompatible scaffolds (for osteoconduction and vascular ingrowth), with appropriate growth factors (for osteoinduction), in order to generate and maintain bone [16]. Therefore, the three prerequisites to consider for engineering bone are the design of an ideal scaffold, the choice of the proper cell type able to differentiate into bone cells, and the regulation of the growth factors/cytokines delivery [17].

A successful 3D scaffold for bone tissue engineering should resemble as much as possible the morphology of natural bone and, in particular, it should mimic the biological structure of bone ECM, which is fundamental for cell adhesion, proliferation and differentiation [18]. Natural bone is a hybrid of inorganic-organic tissue composed of nano-HA crystals (4 nm) and collagen nanofibers (with diameters ranging from 50 to 500 nm), which assembles into a highly porous structure with interconnected pores [19]. Generally, natural bone consists of 60% mineral, 30% collagen and 10% water, with the proportion depending on location and type [20]. The function of collagen fibers is to provide strength in tension and resistance in bending whereas the apatite crystals embedded between the nanofibers resist compression [21]. Since bone is naturally nanostructured, materials with nanometer structure appear to be the best choice for creating bone substitutes. The science dealing with the production of materials with dimensions of less than 100 nm is called nanotechnology, and it is emerging as one of the most powerful engineering approaches [22]. The advantages of nanostructured biomaterials reside in their small size, high porosity, and very importantly in the high surface area-to-volume ratio [23]. In fact, the large surface area of such nanomaterials enhances the adsorption of adhesive proteins (*i.e.*, fibronectin, vitronectin, laminin and collagen), which mediate cell-surface interactions through integrin cell membrane receptors [24].

In recent years, several fabrication methods have been developed for the preparation of nanostructured biomaterials that can be employed as 3D scaffolds in bone tissue engineering. The most used techniques include phase separation, melt-plotting, template synthesis, electrospraying, electrospinning, and the recently developed electrohydrodynamic printing [25]. Through these methods it is possible to generate a variety of structures which can be ordered or random. For example, particles produced by electrospraying or fibers obtained from electrospinning give rise to non-ordered structures with random orientation, which is not a requirement in many clinical applications. However, the use of ordered structures is sometimes essential since the possibility to achieve a better control of certain properties of biomaterials, such as porosity and other mechanical properties, can influence cells behavior. Such aligned structures can be obtained by electrospinning or electrohydrodynamic printing, as described later in the text.

The other important aspect in bone tissue engineering is the introduction of bioactive cells into the 3D-scaffold [26]. In the field of tissue engineering, stem cells are gaining much attention because of their unique characteristics of self-renewal, multi-potency and high potential for *ex vivo* expansion [18]. Mesenchymal stem cells (MSCs) are particularly used in bone tissue engineering because they appear more advantageous than other stem cells. MSCs are multipotent adult stem cells with mesodermal and neuroectodermal origin that can be found in many human tissues, such as bone marrow, adipose tissue, umbilical cord and dental pulp. MSCs can be easily isolated and cultured, and they are able to differentiate into many cell types, including adipocytes, chondrocytes, myocytes and, importantly, osteoblasts [23]. In addition, MSCs do not appear to be rejected by the immune system [26]. In recent years, many efforts have been performed to develop efficient combination of MSCs derived from different sources and appropriate scaffolds for bone tissue reconstruction. This review summarizes the most effective nanostructured biomaterials that are currently used for bone tissue engineering in combination with or without stem cells.

## 2. Materials Used in Bone Tissue Engineering

Metals, ceramics and polymers are the most used biomaterials in bone tissue engineering.

Metals have been widely used as bone replacement materials mainly because of their superior mechanical properties. Nevertheless, metal implants often fail 10–15 years after implantation in the human body as a consequence of a poor and incomplete osteointegration with surrounding bone. Several studies demonstrate that osteointegration is greatly improved with the development of metal implants with nanostructured surfaces. These nanoscaled modifications overall increase surface roughness and wettability, which in turn enhance protein adsorption, cell adhesion, proliferation, and deposition of calcium-containing mineral [22,27,28]. Among all metals, titanium is the material of choice clinically due to its mechanical strength and relatively high degree of biocompatibility. Surface of titanium implants can be modified either by removing material from their surface (subtraction mechanism), or by adding particles on the biomaterial (additive mechanism). The first mechanism includes blasting, acid etching, or grit-blasting followed by acid etching techniques, which have the effect to create pits or pores on the surface of biomaterials. In contrast, the second one generates a surface with bumps by means of titanium plasma-spraying (TPS), or HA and calcium phosphate coatings [29]. This latter modification of titanium surfaces will be described in the section on composite scaffolds.

**Figure 1:**
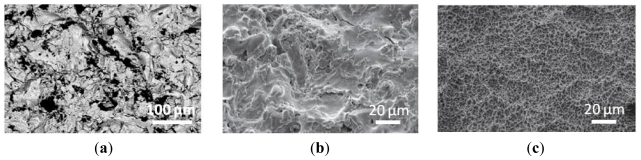
SEM images showing: (**a**) a blasted titanium surface; (**b**) a titanium plasma-spraying (TPS) titanium surface; and (**c**) a sand-blasted large grit-size acid-etched (SLA) titanium surface. Image (**a**) adapted from [30], images (**b**) and (**c**) adapted from [32].

Blasting represents one of the approaches for roughening the titanium surface, and it makes use of hard ceramic particles, such as alumina [30], titanium oxide, and calcium phosphate particles [31]. Figure 1(a) shows an image of alumina blasted titanium surface obtained by scanning electron microscopy (SEM). The ceramic particles are projected into the implants through a nozzle at high velocity by means of compressed air, and different surface roughnesses can be produced on titanium implants according to the size of these ceramic particles.

Another method for roughening titanium implants is the etching with strong acids such as hydrochloric acid (HCl), sulfuric acid (H_2_SO_4_), nitric acid (HNO_3_), and hydrofluoric acid (HF). This method produces micropits on titanium surfaces with sizes ranging from 0.5 to 2 μm in diameter. The immersion of titanium implants in a mixture of concentrated HCl and H_2_SO_4_, the so-called dual acid-etching, promotes rapid osteointegration and enhances the osteoconductive process through the attachment of osteogenic cells [33].

Strong acids are also used in electrochemical anodization. Potentiostatic or galvanostatic anodization of titanium in strong acids at high current density (200 A/m^2^) or potential (100 V) produces micro- and nano-pores on the titanium surface. The anodization process is rather complex and depends on concentration of acids, composition and electrolyte temperature, and current density [29]. Anodized titanium implants in comparison with nonmodified titanium surfaces show more osteointegration, with bone formation occurring directly on these moderately rough surfaces. Conversely, nonmodified titanium surfaces are integrated by the ingrowth of bone from the adjacent bone marrow and preexisting bone tissues [34].

Another approach to rough titanium surfaces derives from the combination of particles blasting with chemical etching, in the so-called sand-blasted large grit-size acid-etched (SLA) method. Wieland and coworkers developed rough surfaces by processing titanium dishes with alumina beads to generate macropits (grit-blasting step) and then eliminating the beads from the SLA surface in the etching step [35]. Surface features produced by the combination of blasting and etching enhanced cell interaction and consequently bone formation. Indeed, osteoblasts seeded on SLA surfaces developed multiple points of attachment that were closely associated with the sub-micrometer features of the surfaces. The cells appeared plated, spread and stretched over the coarse pores and into deep pores, and able to produce bone-like nodules of significantly great sizes. This suggests that roughness in various dimensional ranges could affect osteoblasts differentiation and maturation.

Among the additive mechanisms, TPS represents another strategy to modify the texture surface of titanium implants. This method consists in injecting titanium powders into a plasma torch at high temperature. The titanium particles are projected onto the surface of the implants where they condense and fuse together, forming a uniform coating about 30 μm thick that increases the surface area of the implant. The three-dimensional topography of TPS surface increases the tensile strength at bone-implant contact and, consequently, TPS implants form contact with adjacent bone faster than nonmodified surface implants [29]. In the study of Brett and colleagues, the effects of TPS surfaces on bone cell morphology, attachment, proliferation, and gene expression profile were compared to those of SLA counterparts. These surfaces show little chemical differences but great differences in surface topography, with TPS generating a highly rough titanium surface while SLA producing a moderately rough surface, as shown in Figure 1(b,c), respectively. Cell attachment and proliferation were higher on the TPS surface than on SLA surface, suggesting that increasing degrees of titanium surface roughness elicit enhanced levels of bone cell proliferation *in vitro*. Furthermore, expression profiling analyses showed marked differences in gene responses after 3 h of incubation with cells, which increased further after 24 h, with TPS generating the largest number of up- and down-regulated genes compared with SLA [32]. These findings indicate that the surface roughness of titanium has a profound effect on the profile of genes expressed by bone cells, and suggest that improvements in the biological activity of these materials could be achieved by selective regulation of gene expression through modification of surface roughness.

Also ceramics have been widely used in bone tissue engineering owing to their high biocompatibility with bone cells and tissues, as described earlier. In addition, ceramics are bioactive, which means that they are able to support cell adhesion, proliferation, and differentiation. Calcium orthophosphates (such as HA and TCP) are particularly interesting biomaterials for bone substitutions because of their similarity to the mineral components of human bone. Nevertheless, major limitations to the use of calcium orthophosphates are their brittleness and poor fatigue resistance. In addition, and similar to metals, problems exist regarding insufficient prolonged bonding to the host bone. Recently, Webster and coworkers established that nanosized calcium orthophosphates have relevance in the formation of hard tissues in animals [36]. It has been demonstrated that cell adhesion, proliferation, and calcium deposition is greatly improved on nanophase ceramics compared to micron-sized conventional substrates. This could be explained by the superior wettability of nanoceramics, which promotes a better adsorption of vitronectin, one of the key proteins involved in cell adhesion.

**Figure 2:**
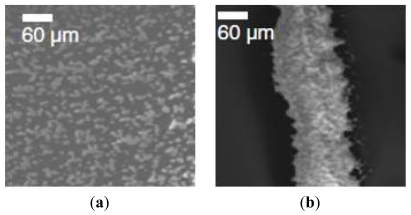
SEM images showing: (**a**) nHA electrospraying obtained with a distance between nozzle and collector of 20 mm; and (**b**) nHA electrohydrodynamic printing generated with a working distance of 0.5 mm. Figure adapted from [40].

One of the nanosized calcium orthophosphates mainly used in bone tissue engineering is nano-hydroxyapatite (nHA). nHA has been produced using a variety of technologies [37–39], among which electrospraying and electrohydrodynamic printing are now gaining more attention. Both of these methods rely on the formation of an electrically-induced jet; however they generate different products since they use different processing parameters, *i.e*., distance, voltage, and flow rate. The main factor influencing the final product is the distance, which is the gap between the nozzle exit and the collecting substrate. Ahmad and colleagues [40] observed that nHA deposition distance >10 mm resulted in spray formation, whereas print formation occurred for distance <3 mm, as shown in Figure 2(a,b), respectively. A transition between electrospraying and electrohydrodynamic printing was visible in this range. The authors also observed that by fixing the printing distance at 0.5 mm and increasing the applied voltage, a variety of transitions were obtained, from dripping, microdripping, rapid micro-dripping, to unstable and stable jetting. The possibility to pattern topographies with diverse morphologies represents a useful way to elicit different cellular responses. For example, Ahmad and coworkers evaluated the behavior of human osteoblast cells (HOBs) seeded on metallic and glass objects coated with electrosprayed- or electrohydrodynamic printed-nHA. Cell behavior on an electrospraying pattern resulted in a random orientation of cells, whereas the cells appeared aligned in the electrohydrodynamic printing pattern.

Polymers are the last group of materials used in bone tissue engineering. Biodegradable polymers can be either natural or synthetic. The natural polymers include polysaccharides (alginate, chitin/chitosan, hyaluronic acid and derivates) or proteins (collagen, fibrin gels, silk). Natural polymers are advantageous because of their high biocompatibility; nevertheless, synthetic polymers have recently attracted growing attention in bone tissue engineering since they can be fabricated to give a wide range of properties and are often free of concerns of immunogenicity [41]. Synthetic polymers most used in bone replacements are poly(L-lactic acid) (PLLA), poly(glycol acid) (PGA), and their copolymers such as poly(lactic-*co*-glycolic acid) (PLGA). It has been demonstrated that the efficacy of polymers is greatly improved through the use of nanotechnology, in a similar way to metals and ceramics. As described above, the reasons reside in the increased adsorption of adhesive proteins on nanophase polymers important for cell attachment [22]. It is now well recognized that arginine-glycine-aspartic acid (RGD) regions in the adsorbed proteins are responsible for the interaction with integrin receptors on cell membrane [41]. Since the biologic action of adhesive proteins is reduced to this RGD sequence, numerous materials including nanopolymers have been RGD functionalized. For example, Paletta and colleagues have shown that the incorporation of RGD peptides into poly(L-lactide) (PLLA) nanofibers has positive effects on cell adhesion and differentiation to some extent [42].

At this point, it is worth saying that polymeric nanofibers are among the best scaffolds for tissue engineering applications, since they reproduce the morphology and structure of the natural ECM, thus providing an ideal setting for cell activities. Currently, there are three common methods for the fabrication of polymeric nanofibers: self-assembly, phase separation and electrospinning [23]. Self-assembly is the most complex technique, and it allows the creation of nanofibers with very small diameters (a few to 100 nm). Phase separation is much simpler than self-assembly and able to generate biocompatible and biodegradable polymers with diameters of 50 to 500 nm. However, these two techniques have the disadvantage to create only short strands of nanofibers. Electrospinning, in contrast, represents the most reliable method to simply fabricate long continuous strands of nanofibers with a diameter ranging from nanometers to microns (50–1,000 nm). In addition, electrospun nanofibers possess the advantages of a very high surface-to-volume ratio and pore sizes ranging from several to tens of micrometers [43]. The electrospinning process is versatile, cost-effective, and there is a wide range of materials that can be spun. Materials used in electrospinning are natural or synthetic biopolymers or their combination, and various substances (HA, proteins, growth factors) can be incorporated into nanofibrous materials [44]. In the electrospinning process, nanofibers are created from a polymeric solution by means of an electrostatic force. The polymeric solution concentration (*c_e_*) represents one of the most influential variables to take into account when producing nanofibers. Indeed, concentrations below *c_e_* produce droplets when the solution is electrified (that are the products of electrospraying). In contrast, above *c_e_*, the electrospun fibers diameter increases with increasing concentration. The *c_e_* value is in turn dependent on the molecular chain length, the chemical nature of the polymer and the solvents selected for the polymer solution. In particular, the choice of a suitable solvent and the development of an appropriate solvent system play a crucial role for the success of nanofibers production. A useful parameter to consider for the selection of the proper solvents and solvent systems is the solubility of the polymer. In the study of Luo and coworkers, 28 different solvents were tested for their solubility and electrospinnability for making 60% w/w polymethylsilsesquioxane (PMSQ) solutions [45]. PMSQ is an interesting hybrid polymer with good thermal stability due to its organic–inorganic nature. It is the highly biocompatible, non-toxic, and chemically stable, properties which make PMSQ a good candidate polymer [46]. In this work, a polymer solution was considered to exhibit good electrospinnability when continuous and stable fiber production with uniform fiber morphology was observed during electrospinning. Surprisingly, solvents of high solubility produced electrospun beads and droplets, which were unsuitable for electrospinning a 60%w/w PMSQ system. Conversely, solvents of partial solubility produced electrospinnable solutions at the same PMSQ concentration. In addition, Luo and colleagues studied suitable binary solvent systems for electrospinning of PMSQ solutions, by combining solvents of different chemical nature, solubility and spinnability. They found that the combination of methanol (high vapor pressure) and propanol (moderate vapor pressure) produced electrospun fibers with high surface porosity, supporting the theory that phase separation can be induced by high vapor pressure of at least one solvent component [47]. Interestingly, the binary solvent system mixing 2-nitropropane (high solubility) and dimethylsulphoxide (non-solvent), neither of which exhibited high volatility, also generated electrospun fibers with high porosity. This demonstrates that phase separation can be induced by solubility difference in the electrospun polymer solution, even if none of the solvent components in the solvent system exhibits high vapor pressure.

**Figure 3:**
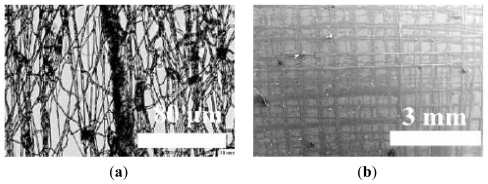
SEM images showing: (**a**) polyurethane (PU) random fibers as a result of electrospinning; and (**b**) PU ordered and overlapped fibers obtained with electrohydrodynamic printing. Figure adapted from [25].

As anticipated earlier in the text, another emerging technique useful for creating fibers and other structures for tissue engineering applications is the electrohydrodynamic printing. This method is very flexible as demonstrated in the work of Ahmad and colleagues, where PMSQ and polyurethane (PU) polymers were used to fabricate either random or ordered structures [25]. For example, by setting appropriate flow rate and voltage needed for a stable jetting, it was possible to produce micrometer-size sparse particles or oriented printed tracks from the same PMSQ solution, only by varying the working distance between the needle and the collector. When using the more viscous PU solution, random fibers with a mean diameter of 3 μm were generated, as reported in Figure 3(a). Also in this case, by adjusting some parameters, more 3D orientated microstructures were produced. Figure 3(b) shows multi-layered pattern of PU with several times of overwriting. These multiple overlapped fibers could provide greater mechanical strength to the overall structure, thus allowing their use as a 3D scaffold material in bone tissue engineering.

## 3. Composite Scaffolds as Bone Substitutes

In recent years, composite biomaterials have been explored for the preparation of bone tissue engineering scaffolds. The major advantage of composites utilization is the creation of new constructs having improved characteristics compared with single components. It is generally accepted the idea that nanocomposites can mimic the constituents of natural bone to some extent better that individual components [48].

Several reports describe the fabrication and the employment of ceramic/metal or ceramic/polymer composites as bone substitutes.

As mentioned above, ceramics are biocompatible with hard human tissues and show good osteoconductive properties. However, their poor mechanical properties limit their applications under load-bearing conditions. On the other hand, mechanical properties of metals are superior for load-bearing implants, but their biocompatibility is much worse than that of ceramics. Studies of Ning and colleagues aimed to combine the bioactivity of HA and the mechanical properties of titanium for fabricating more perfect scaffolds for load-bearing applications [49]. Plasma-sprayed HA coating on the surface of titanium alloys is one of the most extensively reported approaches to achieving this [50,51]. This method relies on HA particles injection into a plasma torch at high temperature and their projection onto the surface of titanium where they condense and fuse together, forming a film. Nevertheless, this method has some disadvantages. First of all, the plasma-spraying determines drastic changes in the composition and crystallinity of the initial calcium phosphate powder; as a consequence, the coating is often not uniform but usually composed of several calcium phosphate phases, such as tricalcium phosphates, tetracalcium phosphate, calcium oxide and amorphous calcium phosphate [52]. Moreover, the HA coating has a tendency to degrade and/or peel off from the titanium implant surface, causing clinical failure of the implant [53]. In order to avoid the drawbacks of plasmasprayed HA coatings, an alternative coating method was developed inspired by the natural process of biomineralization: the precipitation of calcium phosphate apatite crystals onto the titanium surface from simulated body fluids (SBFs) [54,55]. SBFs are fluids with ion concentrations nearly equal those of human blood plasma and are considered the media of choice for evaluating the bioactivity of biomaterials for hard tissue repair [56–58]. In the work of Ning and coworkers, titanium/HA biocomposites were made from titanium and HA powders by powder metallurgy method and their bioactivity was investigated *in vitro* after immersion in SBFs for one week. The bioactivity of a material is defined by its capacity to form bone-bonding with host bone and it can be experimentally predicted from the apatite formation on its surface [59]. The *in vitro* tests of Ning and coworkers showed that apatite formed on the surfaces of titanium/HA composite with higher titanium content (50 and 70%), while no apatite formation was seen on the surfaces of the composite with 30% titanium. This indicates that the *in vitro* bioactivity of the titanium/HA composites is dependent on the initial titanium content. For the *in vivo* experiments, titanium/HA composites in a cylindrical form were implanted in rabbit femur. At the early stage of implantation, the quantity of new bone formed on the composite with 30% titanium was significantly less than that on the surfaces of the 50% and 70% titanium composites. Nevertheless, this difference disappeared six months after implantation. In any case, the titanium/HA composites formed the bone-bonding interface with the surrounding bone through an apatite layer.

Polymers/ceramics represent another type of composite scaffolds. They are widely used as bone tissue substitutes since they mimic to some extent the structure of natural bone ECM. As previously described, bioceramics are ineffective in terms of mechanical stability but they show good osteoconductivity and bone-bonding ability. On the other hand, polymers are biocompatible and degrade into non-toxic components with a controllable degradation rate *in vivo* [60]. Therefore, the combination of these materials can provide scaffolds with good bioactivity, mechanical properties and degradation stability [61]. The potentiality of polymers/ceramics composites in bone regeneration was well confirmed in the study of Lao and colleagues [43]. They showed how PLGA nanofibers mixed with HA particles were able to induce bone mineralization far better than PLGA alone. Therefore, the incorporation of HA particles within PLGA nanofibers could be a meaningful way to increase physical and biological performance of such nanofibrous scaffolds, which are attractive for bone regeneration.

Apart from incorporating ceramics into a biodegradable polymer matrix in the form of fibers, another way to generate polymers/ceramics composites is described in the paper of Nangrejo and colleagues. In this work, co-axial electrohydrodynamic jetting was used for the encapsulation of alumina (the ceramic component) into PMSQ (the polymer counterpart) to produce particles [62]. For this purpose, two suspensions were simultaneously subjected to stable electrohydrodynamic jet flow using concentric needles: the outer needle contained the polymeric solution (30% wt PMSQ in ethanol), whereas the inner needle was filled with the ceramic suspension (10% wt alumina in glycerol). The flow rate of the outer needle was set at twice that of the inner one, and the applied voltage was varied between 0 and 11 kV. At the end of this process, droplets of polymer-coated alumina with particle sizes in the range of 1–38 μm were successfully obtained.

For many years, collagen/HA composites scaffolds have represented the natural choice for bone grafting, due to their similarity to natural bone composition. Both collagen and HA were found to enhance osteoblast differentiation, but, when combined together, they were shown to accelerate osteogenesis [63]. Venugopal and coworkers produced electrospun nanofibers with collagen/HA (1:1) using 1,1,1,3,3,3-hexafluoro propanol (HFP) as solvent [64]. Human fetal osteoblast cells (hFOBs) were then seeded onto these fibers for evaluating their response in terms of proliferation and mineralization, when compared to a nanofibrous scaffold made of collagen alone. Mineralization refers to cell-mediated deposition of extracellular calcium and phosphorus salts where anionic matrix molecules take up the Ca^2+^ and PO_4_^3−^ ions and serve as nucleation and growth sites, leading to calcification [65]. Mineralization is generally quantified by the alizarin red-S (ARS) staining. ARS is a dye which binds selectively calcium salts and it is widely used for calcium mineral histochemistry. After 10 days of culturing, both electrospun nanofibrous scaffolds showed a comparable rate of hFOBs proliferation. Nevertheless, the mineral deposition on collagen/HA nanofibrous scaffolds cultured with osteoblasts was much higher than that on collagen nanofibers alone. This work showed that the collagen/HA composite nanofibrous scaffolds have great potential for bone tissue regeneration. Some limitations however exist when working with collagen, as it is costly, it degrade rapidly in the biological environment, and may have problems related to its antigenicity [66]. For this reason, subsequent studies have explored the possibility of replacing collagen with other natural biopolymers in order to prepare HA-containing electrospun composite fibers for potential osteoregenerative applications.

Gelatin, for example, is sometimes used instead of collagen. It is a hydrolyzed form of collagen extracted from skin, bone, tendon, ligament, and other connective tissues [67]. Since gelatin is a denatured biopolymer, the selection of gelatin as a scaffolding material can circumvent the concerns of immunogenicity and pathogen transmission associated with collagen. Gelatin contains integrin binding sites for cell adhesion and carboxylic acid groups that bind calcium ions present in HA [68]. In the work of Francis and coworkers, gelatin was used to produce nanofibrous scaffolds in combination with nHA [69]. In particular, four different formulations were prepared and compared: gelatin, gelatin/HA (4:1 blend), gelatin/HA (2:1 blend) and gelatin/HA (spin–spray) nanofibers. The difference between the blended and the spin–spray matrices is that in the first kind of scaffold HA nanoparticles are distributed inside the gelatin nanofibers, whereas in the second one, nHA particles are highly dispersed on the surface of the nanofibers. All these electrospun scaffolds were then subjected to chemical cross-linking with glutaraldehyde (GA) vapors. Cross-linking is necessary to maintain the structural integrity of the scaffold during the desired implantation time, before cells repopulate and new tissue regenerates [70]. Before evaluating the *in vitro* biocompatibility of these scaffolds, field emission scanning electron microscopy (FESEM) analyses were performed in order to morphologically characterize these fibers. Electrospun fiber diameters were observed in the ranges of 589 ± 103, 453 ± 84, 421 ± 75 and 679 ± 147 nm, respectively, for gelatin, gelatin/HA (4:1 blend), gelatin/HA (2:1 blend) and gelatin/HA (spin–spray) nanofibers. After cross-linking with GA vapors, the average fiber diameters increased to 750 ± 160, 555 ± 116, 501 ± 102 and 784 ± 120 nm, respectively, for the above listed scaffolds. This increment could be attributed to swelling of nanofibers during the cross-linking process, and it was more consistent for the spin–spray nanofibrous scaffolds, probably because of the separate dispersion of nanoparticles during the electrospraying process. At this point, hFOBs cells were cultured onto the 4 different scaffolds. With increasing culture time, cell proliferation was found to be significantly higher on the spin-spray gelatin/HA scaffolds than on the blended counterparts. In addition, also the alkaline phosphatase (ALP) activity was found to increase with time and it was higher for the gelatin/HA scaffolds obtained with the spin-spray method than blended scaffolds. ALP is a well-known enzyme able to catalyze the hydrolysis of phosphate group, thus increasing the local phosphate concentration and enhancing the mineralization of ECM. For this reason, ALP is considered an early-stage marker of the osteogenic phenotype and its elevated activity is observed before the initiation of calcium minerals [10]. Mineralization was quantified by the ARS staining. The gelatin/HA spin–spray scaffolds showed intensely stained images, indicating more calcium deposition than gelatin and gelatin/HA blend scaffolds. In the hFOBs mineralization process, cell adhesion was enhanced by the gelatin polymer matrix and mineral deposition was enhanced by HA in gelatin/HA scaffolds. Taken together, the results of Francis and colleagues clearly demonstrate that the complete exposure of nHA in the spin–spray nanofibers provides a scaffold with superior osteoconductive and osteoinductive properties, leading to a better attachment, proliferation and mineralization of hFOB cells.

Chitosan represents another alternative to collagen for the production of chitosan/HA composite scaffolds usable in bone tissue engineering. Chitosan is a natural biopolymer derived from deacetylation of chitin, which is the structural element in the exoskeleton of crustaceans (such as crabs and shrimp) and cell walls of fungi. Chitosan is known for its biodegradability, biocompatibility, low immunogenicity, and excellent mechanical properties. However, the poor electrospinnability of the chitosan itself, together with the adverse effect of the non-electrospinnable HA nanoparticles (and their aggolmerates) make difficult the conversion of chitosan/HA nanocomposites into a fibrous form by electrospinning. In order to overcome these complications, Zhang and coworkers developed a two-step method for producing chitosan/HA nanofibrous scaffolds for bone tissue engineering [71]. They firstly prepared chitosan/HA nanocomposites (70:30 in mass ratio) by a co-precipitation synthesis approach, which allowed a better dispersion of nHA into the chitosan matrix. Then, an ultrahigh molecular weight poly(ethylene oxide) (UHMWPEO) was used to substantially aid the formation of chitosan nanofibrous structure via the electrospinning process. An aqueous acetic acid solution was used as dominant solvent system, which enabled proper structural preservation of HA crystallites. The electrospun chitosan/HA nanocomposite fibers appeared continuous and geometrically uniform with a diameter of 214 ± 25 nm, as resulted by FESEM analyses. hFOBs were then cultured for up to 15 days onto these scaffolds. After 5 days of seeding, hFOBs proliferation on the chitosan/HA nanofibrous scaffold was low and comparable with that of the pure electrospun chitosan matrix, but it significantly increased by days 10 and 15. The authors speculated that the presence of the UHMWPEO on the fibers surface probably reduced protein adsorption, therefore delaying cells adhesion and proliferation. However, this inhibition effect disappeared in concomitance with the gradual solubility of UHMWPEO in the culture medium, resulting in an increased level of cell proliferation with time. In addition to cell proliferation, also mineralization resulted higher for the composite scaffolds at day 15 as demonstrated by the ARS staining. These results overall demonstrated that despite an initial inhibition, the osteoconductive effect of the incorporated HA nanoparticles stimulated a more significant level of bone cell formation ability when using the composite nanofibers of chitosan/HA scaffolds.

Another example of chitosan/HA composite nanofibrous scaffold comes from the recent work of Venugopal and colleagues [72]. They prepared chitosan/HA (80:25) electrospun nanofibers by dissolving in trifluoroacetic acid/dichloromethane (TFA/DCM) (70:30 w/w), and evaluated the proliferation and mineralization of hFOBs in comparison to a chitosan alone nanofibrous scaffold. Also in this case, the excellent osteoconductivity of HA in the composite scaffolds notably enhanced the bone forming ability as shown by cell proliferation, mineral deposition, and morphology observation.

Also synthetic polymers are now being widely used as scaffolds in combination with HA in bone tissue engineering. As anticipated earlier, the main advantage of most of the synthetic biodegradable polymers studied to date are the absence of immunogenicity or disease transmission. In addition, characteristics such as strength, degradability, and adhesiveness can be altered to facilitate their clinical use [41]. In a recent work of Peng and colleagues [19], HA/PLLA nanofibrous scaffolds were fabricated as random or aligned assemblies. Both nano- or micro-sized HA (nHA or mHA) particles were incorporated into PLLA nanofibers with a diameter of about 300 nm using electrospinning. The bioactivity and cell signaling properties of these scaffolds were evaluated by *in vitro* culturing rat osteosarcoma cells onto the scaffold surfaces for 10 days. All composite scaffolds supported cell adhesion, proliferation and differentiation, but to different extent. The mHA/PLLA random scaffold showed greater cell proliferation than nHA/PLLA random scaffold, whereas both the composite aligned scaffolds supported cell proliferation at a similar level. However, no significant differences were observed between the random and aligned composite scaffolds. Similar results were obtained by measuring the ALP activity on each scaffold. The random and aligned fibrous assemblies had rather a pronounced effect on the morphology of the cells in direct contact with the scaffold surface. The cells on the random scaffolds showed multipolar extensions and had a polygonal and flat shape; in contrast, most cells on aligned scaffolds exhibited dipolar extensions and a spindle shape. In general, HA/PLLA nanofibrous scaffolds seem to be good candidates for bone tissue engineering.

**Figure 4:**
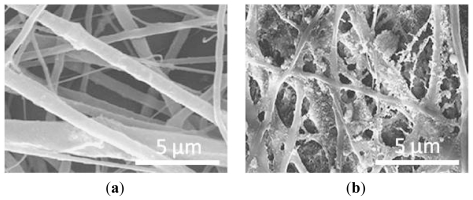
FESEM images of (**a**) electrospun PLACL/gelatin/HA-blended nanofibers and (**b**) electrospun PLACL/gelatin/HA-sprayed nanofibers. Figure adapted from [73].

To improve the properties of polymeric biomaterials, composites made from synthetic/natural polymers and ceramics have been investigated extensively for bone replacements. Gupta and colleagues, for example, produced composite nanofibers starting from poly(L-lactic acid)-co-poly(3-caprolactone) (PLACL), gelatin and HA nanoparticles [73]. PLACL is a synthetic, biodegradable, non-toxic copolymer of PLLA and poly(3-caprolactone) (PCL) [74]. PLLA and PCL have different biodegradability rates and have been used for different applications in tissue engineering. Their copolymer, PLACL, has already been used as substrate for culturing smooth muscle and endothelial cells [75–77]. In the study of Gupta and coworkers, simultaneous electrospraying and electrospinning techniques were used to generate PLACL/gelatin/HA nanofibers, and their effect on hFOBs proliferation and mineralization was evaluated in comparison to electrospun PLACL/gelatin/HA-blended nanofibers. FESEM analyses revealed that electrospun PLACL/gelatin/HA-blended nanofibers showed mean diameters of 198 ± 107 nm, whereas fiber diameters of electrosprayed PLACL/gelatin/HA nanofibers were 406 ± 155 nm. These increased fiber sizes of PLACL/gelatin/HA-sprayed scaffolds could probably be explained by the formation of a layer on surface of these polymer fibers, as a consequence of the spraying of HA nanoparticles on and around the fibers. Figure 4(a,b) well evidences the morphological difference between blended and sprayed electrospun nanofibers composites. After 15 days of cell seeding, a significant increase in proliferation was seen on PLACL/gelatin/HA-sprayed scaffolds with respect to HA-blended scaffolds, as well as an enhanced ALP activity and mineralization. As illustrated before, the superior performance of electrosprayed-HA nanoparticles could be explained by the complete exposure of nHA on the nanofibers surfaces, which in turn augmented their osteoinductive and osteoconductive properties.

Apart from an appropriate scaffold, a successful system for bone tissue engineering also requires the presence of bioactive cells able to regenerate the damaged tissue. In recent years, the abundance and accessibility of MSCs may prove to be novel cell therapeutics for bone repair and regeneration. Encouraging results came from the study of Wang and coworkers [26], where nHA/polyamide (nHA/PA) composite matrices were fabricated by phase-inversion and used as scaffolds for seeding bone marrow-derived MSCs. For the *in vitro* experiments, MSC/scaffold constructs were cultured for 7 days. nHA/PA composites resulted biocompatible and non-cytotoxic to cells, with no negative effect on MSCs adhesion and proliferation. The ALP activity and type I collagen immunostaining were used to quantitatively and qualitatively determine the differentiation of MSCs into osteoblastic phenotype. Like ALP, type I collagen is another important marker whose expression increases during the early stage of osteogenic differentiation. Both tests gave positive results, thus indicating that scaffolds positively influenced MSCs osteoblastic differentiation. These findings were confirmed by *in vivo* experiments where pure nHA/PA scaffolds and MSC/scaffold constructs were implanted in rabbit mandibles and studied histologically and microradiografically. Their results showed that pure nHA/PA scaffolds exhibited good biocompatibility and osteoconductivity with host bone. However, the introduction of MSCs into the composite scaffold considerably enhanced the efficiency of new bone formation, in particular at early stages after implantation. Nevertheless, in long-term experiments, both the pure scaffolds and MSC/scaffold constructs exhibited good biocompatibility and osteoconductivity.

In terms of osteoinductive growth factors, most research has focused on the use of the BMPs. BMPs belong to the transforming growth factor-beta (TGF-β) family and are considered one of the most potent factors promoting bone growth. In particular, BMP-7 appears to be one of the strong osteoinductive molecules that can stimulate new bone formation [78]. In the recent work of Li and colleagues [79], MSCs were transfected with BMP-7, seeded on nHA/PA porous scaffolds and then implanted in rabbit mandibular defects. To evaluate new bone formation, radiographical, biomechanical, histological, and histomorphometrical analyses were performed after different implantation periods. At early stages after implantation, scaffolds seeded with BMP-7 transfected MSCs showed a faster response compared to MSCs/scaffolds and pure nHA/PA scaffolds. However, no difference in osteointegration was found among the three groups at 16-week postimplantation. This study highlighted the relevance of factors and cells in accelerating bone formation during the early stages after implantation.

**Figure 5:**
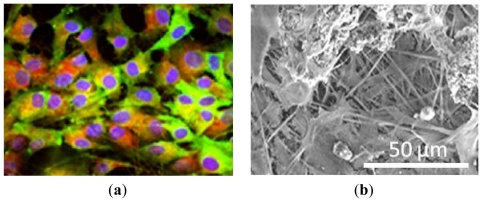
(**a**) Confocal microscopy image of PLLA/PBLG/collagen/HA nanofibers showing osteogenic differentiation of ADSCs, expressing both CD105 (green) and OCN (red); 60× magnification; (**b**) SEM image showing minerals secreted by ADSCs on PLLA/PBLG/collagen/HA nanofibers; 2,500× magnification. Figure adapted from [80].

An interesting work has been described very recently by Ravichandran and colleagues, who were able to guide the osteogenic differentiation of MSCs isolated from adipose tissue (adipose-derived stem cells, ADSCs) in the absence of any induction medium. For this purpose, they functionalized the surfaces of PLLA/collagen electrospun nanofibers with poly-benzyl-L-glutamate (PBLG) [80]. PBLG is a polymer of glutamic acid in which the γ-carboxyl groups have been benzoylated. In addition, derivatives of glutamic acid like PBLG possess high calcium binding affinity, which is essential for differentiation and bone regeneration. HA nanoparticles were then deposited onto electrospun PLLA/PBLG/collagen fibers by calcium-phosphate dipping method in order to assure good osteoconductive properties at the scaffolds. Electrospun nanofibers made of PLLA, PLLA/PBLG, or PLLA/PBLG/collagen were used as controls. Morphological analyses performed by SEM revealed that the fiber diameters of all the electrospun nanofibers were in the nanometer range, varying from 200 to 400 nm. The capacity of nanofiber scaffolds to support ADSCs adhesion and proliferation was evaluated using cell proliferation assay. After 14 and 21 days of seeding, the rate of proliferation was the highest on PLLA/PBLG/collagen/HA scaffolds compared to other scaffolds. The authors speculated that the presence of the adhesive protein PBLG made possible the interaction with the integrins on the surface of ADSCs, thus contributing to a better cell proliferation compared to scaffolds lacking in any cell binding. Also the presence of HA nanoparticles positively affected ADSCs proliferation, by providing a greater surface area for more cells adhesion onto the scaffold. The four different scaffolds were then compared for the ALP activity, which resulted higher on the PLLA/PBLG/collagen/HA nanofibers on day 7, 14 and 21. Nevertheless, there was no significant increase in the ALP activity from day 14–21 onto such scaffolds, probably indicating a switch to the mineralization phase, which in turn demonstrates ADSCs differentiation towards the osteoblastic phenotype. The mineral deposition was evaluated by means of ARS staining, resulting much higher on the PLLA/PBLG/collagen/HA scaffolds compared to other nanofiber constructs on day 14 and 21. The osteogenic differentiation of ADSCs was also confirmed by dual immunofluorescent staining with CD105, an ADSC specific marker protein, and osteocalcin (OCN), an osteoblasts specific marker ECM protein. It is well known that OCN plays a significant role in modulating mineralization, as it has glutamic acid rich regions with strong binding affinities to both Ca^2+^ and HA [81]. The complete osteogenic differentiation of ADSCs seeded onto PLLA/PBLG/collagen/HA nanofibers was supported by their cuboidal morphology, which is a typical characteristic of mature osteoblasts, as demonstrated by immunostaining in Figure 5(a). Further SEM analyses revealed how ADSCs on PLLA/PBLG/collagen/HA nanofibers produced much more mineral deposit and aggregated into large mineral clumps with respect to the other scaffolds, as shown in Figure 5(b). The very important aspect of this study is represented by the introduction of the bioactive PBLG and HA molecules on the electrospun nanofibers, which are able to regulate and improve adhesion, proliferation and differentiation of ADSCs into osteogenic lineage.

## 4. Conclusions

In recent years, rapid developments in nanotechnology have yielded many clinical benefits, in particular in the field of bone tissue engineering. The main advantage is that several novel biomaterials can be fabricated into nanostructures that closely mimic the bone in structure and composition. The optimization in the surface features of biomaterials has strongly improved cell behavior in terms of adhesion, proliferation, differentiation and tissue formation in three dimensions. Such advances were previously unimaginable with conventional materials possessing large micron-sized particles. Nevertheless, further studies need to be done in order to identify the best combination of biomaterials, cells and engineering approaches for creating the appropriate system for specific medical applications. To achieve this goal, the effective cooperation of clinicians, biologists, chemists, bioengineers and materials scientists will be required.

## References

[1] Swetha M., Sahithi K., Moorthi A., Srinivasan N., Ramasamy K., Selvamurugan N. (2010). Biocomposites containing natural polymers and hydroxyapatite for bone tissue engineering. Int. J. Biol. Macromol.

[2] Lee K.J., Roper J.G., Wang J.C. (2005). Demineralized bone matrix and spinal arthrodesis. Spine J.

[3] Giannoudis P.V., Dinopoulos H., Tsiridis E. (2005). Bone substitutes: An update. Injury.

[4] Summers B.N., Eisenstein S.M. (1989). Donor site pain from the ilium. A complication of lumbar spine fusion. J. Bone Joint Surg. Br.

[5] Arrington E.D., Smith W.J., Chambers H.G., Bucknell A.L., Davino N.A. (1996). Complications of iliac crest bone graft harvesting. Clin. Orthop. Relat. Res.

[6] Seiler J.G., Johnson J. (2000). Iliac crest autogenous bone grafting: Donor site complications. J. South. Orthop. Assoc.

[7] Sandhu H.S., Grewal H.S., Parvataneni H. (1999). Bone grafting for spinal fusion. Orthop. Clin. North Am.

[8] Ehrler D.M., Vaccaio A.R. (2000). The use of allograft bone in lumbar spine surgery. Clin. Orthop. Relat. Res.

[9] Finkemeier C.G. (2002). Bone-grafting and bone-graft substitutes. J. Bone Joint Surg. Am.

[10] Duan B., Wang M., Zhou W.Y., Cheung W.L., Li Z.Y., Lu W.W. (2010). Three-dimensional nanocomposite scaffolds fabricated via selective laser sintering for bone tissue engineering. Acta Biomater.

[11] Damien C.J., Parsons J.R. (1991). Bone graft and bone graft substitutes: A review of current technology and applications. J. Appl. Biomater.

[12] Parikh S.N. (2002). Bone graft substitutes: Past, present, future. J. Postgrad. Med.

[13] Heise U., Osborn J.F., Duwe F. (1990). Hydroxyapatite ceramic as a bone substitute. Int. Orthop.

[14] Kinnunen I., Aitasalo K., Pöllönen M., Varpula M. (2000). Reconstruction of orbital floor fractures using bioactive glass. J. Craniomaxillofac. Surg.

[15] Meyer U., Joos U., Wiesmann H.P. (2004). Biological and biophysical principles in extracorporal bone tissue engineering. Part III. Int. J. Oral Maxillofac. Surg.

[16] Jones E.A., Yang X.B. (2005). Mesenchymal stem cells and their future in bone repair. Int. J. Adv. Rheumatol.

[17] Chen J.P., Chang Y.S. (2011). Preparation and characterization of composite nanofibers of polycaprolactone and nanohydroxyapatite for osteogenic differentiation of mesenchymal stem cells. Colloids Surf. B Biointerfaces.

[18] Seyedjafari E., Soleimani M., Ghaemi N., Sarbolouki M.N. (2011). Enhanced osteogenic differentiation of cord blood-derived unrestricted somatic stem cells on electrospun nanofibers. J. Mater. Sci. Mater. Med.

[19] Peng F., Yu X., Wei M. (2011). *In vitro* cell performance on hydroxyapatite particles/poly(L-lactic acid) nanofibrous scaffolds with an excellent particle along nanofiber orientation. Acta Biomater.

[20] Athanasiou K.A., Zhu C., Lanctot D.R., Agrawal C.M., Wang X. (2000). Fundamentals of biomechanics in tissue engineering of bone. Tissue Eng.

[21] Ma J., He X., Jabbari E. (2011). Osteogenic differentiation of marrow stromal cells on random and aligned electrospun poly(L-lactide) nanofibers. Ann. Biomed. Eng.

[22] Tran N., Webster T.J. (2009). Nanotechnology for bone materials. Wiley Interdiscip. Rev. Nanomed. Nanobiotechnol.

[23] Ngiam M., Nguyen L.T., Liao S., Chan C.K., Ramakrishna S. (2011). Biomimetic nanostructured materials: Potential regulators for osteogenesis?. Ann. Acad. Med. Singap.

[24] Kubinová S., Syková E. (2010). Nanotechnologies in regenerative medicine. Minim. Invasive Ther. Allied Technol.

[25] Ahmad Z., Rasekh M., Edirisinghe M. (2010). Electrohydrodynamic direct writing of biomedical polymers and composites. Macromol. Mater. Eng.

[26] Wang H., Li Y., Zuo Y., Li J., Ma S., Cheng L. (2007). Biocompatibility and osteogenesis of biomimetic nano-hydroxyapatite/polyamide composite scaffolds for bone tissue engineering. Biomaterials.

[27] Wall I., Donos N., Carlqvist K., Jones F., Brett P. (2009). Modified titanium surfaces promote accelerated osteogenic differentiation of mesenchymal stromal cells *in vitro*. Bone.

[28] Mendonça G., Mendonça D.B., Aragão F.J., Cooper L.F. (2010). The combination of micron and nanotopography by H(2)SO(4)/H(2)O(2) treatment and its effects on osteoblast-specific gene expression of hMSCs. J. Biomed. Mater. Res. A.

[29] le Guéhennec L., Soueidan A., Layrolle P., Amouriq Y. (2007). Surface treatments of titanium dental implants for rapid osseointegration. Dent. Mater.

[30] Aparicio C., Gil F.J., Fonseca C., Barbosa M., Planell J.A. (2003). Corrosion behaviour of commercially pure titanium shot blasted with different materials and sizes of shot particles for dental implant applications. Biomaterials.

[31] Müeller W.D., Gross U., Fritz T., Voigt C., Fischer P., Berger G., Rogaschewski S., Lange K.P. (2003). Evaluation of the interface between bone and titanium surfaces being blasted by aluminium oxide or bioceramic particles. Clin. Oral Implants Res.

[32] Brett P.M., Harle J., Salih V., Mihoc R., Olsen I., Jones F.H., Tonetti M. (2004). Roughness response genes in osteoblasts. Bone.

[33] Trisi P., Lazzara R., Rebaudi A., Rao W., Testori T., Porter S.S. (2003). Bone-implant contact on machined and dual acid-etched surfaces after 2 months of healing in the human maxilla. J. Periodontol.

[34] Burgos P.M., Rasmusson L., Meirelles L., Sennerby L. (2008). Early bone tissue responses to turned and oxidized implants in the rabbit tibia. Clin. Implant Dent. Relat. Res.

[35] Wieland M., Textor M., Chehroudi B., Brunette D.M. (2005). Synergistic interaction of topographic features in the production of bone-like nodules on Ti surfaces by rat osteoblasts. Biomaterials.

[36] Webster T.J., Schadler L.S., Siegel R.W., Bizios R. (2001). Mechanisms of enhanced osteoblast adhesion on nanophase alumina involve vitronectin. Tissue Eng.

[37] Halloran J.W. (1999). Freeform fabrication of ceramics. Br. Ceram. Trans.

[38] Yang Y., Kim K.H., Ong J.L. (2005). A review on calcium phosphate coatings produced using a sputtering process-an alternative to plasma spraying. Biomaterials.

[39] Liang H., Shi B., Fairchild A. (2004). Applications of plasma coatings in artificial joints: An overview. Vacuum.

[40] Ahmad Z., Thian E.S., Huang J., Edirisinghe M.J., Best S.M., Jayasinghe S.N., Bonfield W., Brooks R.A., Rushton N. (2008). Deposition of nano-hydroxyapatite particles utilising direct and transitional electrohydrodynamic processes. J. Mater. Sci. Mater. Med.

[41] Balasundaram G., Webster T.J. (2007). An overview of nano-polymers for orthopedic applications. Macromol. Biosci.

[42] Paletta J.R., Bockelmann S., Walz A., Theisen C., Wendorff J.H., Greiner A., Fuchs-Winkelmann S., Schofer M.D. (2010). RGD-functionalisation of PLLA nanofibers by surface coupling using plasma treatment: Influence on stem cell differentiation. J. Mater. Sci. Mater. Med.

[43] Lao L., Wang Y., Zhu Y., Zhang Y., Gao C. (2011). Poly(lactide-co-glycolide)/hydroxyapatite nanofibrous scaffolds fabricated by electrospinning for bone tissue engineering. J. Mater. Sci. Mater. Med.

[44] Sun F., Zhou H., Lee J. (2011). Various preparation methods of highly porous hydroxyapatite/polymer nanoscale biocomposites for bone regeneration. Acta Biomater.

[45] Luo C.J., Nangrejo M., Edirisinghe M. (2010). A novel method of selecting solvents for polymer electrospinning. Polymer.

[46] Ma J., Shi L., Shi Y., Luo S., Xu J. (2002). Pyrolysis of polymethylsilsesquioxane. J. Appl. Polym. Sci.

[47] Megelski S., Stephens J.S., Chase D.B., Rabolt J.F. (2002). Micro- and nanostructured surface morphology on electrospun polymer fibers. Macromolecules.

[48] Liu H., Slamovich E.B., Webster T.J. (2006). Increased osteoblast functions among nanophase titania/poly(lactide-co-glycolide) composites of the highest nanometer surface roughness. J. Biomed. Mater. Res. A.

[49] Ning C., Zhou Y. (2008). Correlations between the *in vitro* and *in vivo* bioactivity of the Ti/HA composites fabricated by a powder metallurgy method. Acta Biomater.

[50] de Groot K., Geesink R., Klein C.P.A.T., Serekian P. (1987). Plasma sprayed coatings of hydroxyapatite. J. Biomed. Mater. Res.

[51] Yang Y.C. (2007). Influence of residual stress on bonding strength of the plasma-sprayed hydroxyapatite coating after the vacuum heat treatment. Surf. Coat. Technol.

[52] Radin S.R., Ducheyne P. (1992). Plasma spraying induced changes of calcium phosphate ceramic characteristics and the effect on *in vitro* stability. J. Mater. Sci. Mater. Med.

[53] Lee J.J., Rouhfar L., Beirne O.R. (2000). Survival of hydroxyapatite-coated implants: A meta-analytic review. J. Oral Maxillofac. Surg.

[54] Wang X., Yan W., Hayakawa S., Tsuru K., Osaka A. (2003). Apatite deposition on thermally and anodically oxidized titanium surfaces in a simulated body fluid. Biomaterials.

[55] Barrere F., Snel M., van Blitterswijk C., de Groot K., Layrolle P. (2004). Nano-scale study of the nucleation and growth of calcium phosphate coating on titanium implants. Biomaterials.

[56] Kokubo T., Kushitani H., Sakka S., Kitsugi T., Yamamuro T. (1990). Solutions able to reproduce *in vivo* surface-structure changes in bioactive glass-ceramic A-W. J. Biomed. Mater. Res.

[57] Kokubo T., Takadama H. (2006). How useful is SBF in predicting *in vivo* bone bioactivity?. Biomaterials.

[58] Rasekh M., Ahmad Z., Day R., Wickam A., Edirisinghe M. (2011). Direct Writing of Polycaprolactone Polymer for Potential Biomedical Engineering Applications. Adv. Eng. Mater.

[59] Hench L.L. (1998). Biomaterials: A forecast for the future. Biomaterials.

[60] Wei G., Ma P.X. (2004). Structure and properties of nano-hydroxyapatite/polymer composite scaffolds for bone tissue engineering. Biomaterials.

[61] Jose M.V., Thomas V., Xu Y., Bellis S., Nyairo E., Dean D. (2010). Aligned bioactive multi-component nanofibrous nanocomposite scaffolds for bone tissue engineering. Macromol. Biosci.

[62] Nangrejo M., Ahmad Z., Edirisinghe M. (2010). Ceramic encapsulation with polymer via co-axial electrohydrodynamic jetting. J. Microencapsul.

[63] Xie J., Baumann M.J., McCabe L.R. (2004). Osteoblasts respond to hydroxyapatite surfaces with immediate changes in gene expression. J. Biomed. Mater. Res. A.

[64] Venugopal J., Low S., Choon A.T., Sampath Kumar T.S., Ramakrishna S. (2008). Mineralization of osteoblasts with electrospun collagen/hydroxyapatite nanofibers. J. Mater. Sci. Mater. Med.

[65] Boskey A.L. (1998). Biomineralization: Conflict, challenges and opportunities. J. Cell. Biochem. Suppl.

[66] Venugopal J., Prabhakaran M.P., Zhang Y., Low S., Choon A.T., Ramakrishna S. (2010). Biomimetic hydroxyapatite-containing composite nanofibrous substrates for bone tissue engineering. Philos. Trans. A Math. Phys. Eng. Sci.

[67] Harrington W.F., Vonhippel P.H. (1961). The structure of collagen and gelatin. Adv. Protein Chem.

[68] Jacobson R.J., Brown L.L., Hutson T.B., Fink D.J., Veis A. (1983). Intermolecular interactions in collagen self assembly as revealed by fourier transform infrared spectroscopy. Science.

[69] Francis L., Venugopal J., Prabhakaran M.P., Thavasi V., Marsano E., Ramakrishna S. (2010). Simultaneous electrospin-electrosprayed biocomposite nanofibrous scaffolds for bone tissue regeneration. Acta Biomater.

[70] Pieper J.S., Hafmans T., Veerkamp J.H., van Kuppevelt T.H. (2000). Development of tailor-made collagen-glycosaminoglycan matrices: EDC/NHS crosslinking, and ultrastructural aspects. Biomaterials.

[71] Zhang Y., Venugopal J.R., El-Turki A., Ramakrishna S., Su B., Lim C.T. (2008). Electrospun biomimetic nanocomposite nanofibers of hydroxyapatite/chitosan for bone tissue engineering. Biomaterials.

[72] Venugopal J.R., Giri Dev V.R., Senthilram T., Sathiskumar D., Gupta D., Ramakrishna S. (2011). Osteoblast mineralization with composite nanofibrous substrate for bone tissue regeneration. Cell Biol. Int.

[73] Gupta D., Venugopal J., Mitra S., Giri Dev V.R., Ramakrishna S. (2009). Nanostructured biocomposite substrates by electrospinning and electrospraying for the mineralization of osteoblasts. Biomaterials.

[74] Lemmouchi Y., Schatch E. (1997). Preparation and *in vitro* evaluation of biodegradable poly(3-caprolactone-co-D,L lactide) (X–Y) devises containing tryparocidal drugs. J. Control Release.

[75] Mo X.M., Xu X.Y., Kotaki M., Ramakrishna S. (2004). Electrospun P(LLA-CL) nanofiber: A biomimetic extracellular matrix for smooth muscle cells and endothelial proliferation. Biomaterials.

[76] Xu X.Y., Inai R., Kotaki M., Ramakrishna S. (2004). Aligned biodegradable nanofibrous structure: A potential scaffold for blood vessel engineering. Biomaterials.

[77] He W., Yong T., Ma Z., Inai R., Teo W.E., Ramakrishna S. (2006). Biodegradable polymer nanofiber mesh to maintain functions of endothelial cells. Tissue Eng.

[78] Yang M., Ma Q.J., Dang G.T., Ma K., Chen P., Zhou C.Y. (2005). *In vitro* and *in vivo* induction of bone formation based on *ex vivo* gene therapy using rat adipose-derived adult stem cells expressing BMP-7. Cytotherapy.

[79] Li J., Li Y., Ma S., Gao Y., Zuo Y., Hu J. (2010). Enhancement of bone formation by BMP-7 transduced MSCs on biomimetic nano-hydroxyapatite/polyamide composite scaffolds in repair of mandibular defects. J. Biomed. Mater. Res. A.

[80] Ravichandran R., Venugopal J.R., Sundarrajan S., Mukherjee S., Ramakrishna S. (2012). Precipitation of nanohydroxyapatite on PLLA/PBLG/Collagen nanofibrous structures for the differentiation of adipose derived stem cells to osteogenic lineage. Biomaterials.

[81] Young M.F., Kerr J.M., Ibaraki K., Heegaard A.M., Robey P.G. (1992). Structure, expression, and regulation of the major noncollagenous matrix proteins of bone. Clin. Orthop. Relat. Res.

