# Correction: Nanostructured Biomaterials for Tissue Engineered Bone Tissue Reconstruction. *Int. J. Mol. Sci.* 2012, *13*, 737–757

**DOI:** 10.3390/ijms13056452

**Published:** 2012-05-24

**Authors:** Chiara Gardin, Letizia Ferroni, Lorenzo Favero, Edoardo Stellini, Dario Stomaci, Stefano Sivolella, Eriberto Bressan, Barbara Zavan

**Affiliations:** 1 Department of Histology, Microbiology and Medical Biotechnology, University of Padova, Via G. Colombo 3, 35100 Padova, Italy; E-Mails: chiara.gardin@unipd.it (C.G.); letizia.ferroni@unipd.it (L.F.); 2 Department of Periodontology, School of Dentistry, University of Padova, Via Venezia 90, 35100 Padova, Italy; E-Mails: lorenzo.favero@unipd.it (L.F.); edoardo.stellini@unipd.it (E.S.); dstomaci@yahoo.it (D.S.); stefano.sivolella@libero.it (S.S.); eriberto.bressan@unipd.it (E.B.)

We would like to change the authors’ names and E-Mail addresses on Page 737 of Article [1] from:

**Gardin Chiara** **^1^, Ferroni Letizia** **^1^, Favero Lorenzo** **^2^, Stellini Edoardo** **^2^, Stomaci Diego** **^2^, Sivolella Stefano** **^2^, Bressan Eriberto** **^2^** **and Zavan Barbara** ^1,*^

^1^ Department of Histology, Microbiology and Medical Biotechnology, University of Padova, Via G. Colombo 3, 35100 Padova, Italy; E-Mails: Gardinc@unipd.it (G.C.); Ferronil@unipd.it (F.L.)

^2^ Department of Periodontology, School of Dentistry, University of Padova; Via Venezia 90, 35100 Padova, Italy; E-Mails: Stomacid@unipd.it (S.D.); eribertobresan@unipd.it (B.E.)

To the correct version, as follows:

**Chiara Gardin** **^1^, Letizia Ferroni** **^1^, Lorenzo Favero** **^2^, Edoardo Stellini** **^2^, Dario Stomaci** **^2^, Stefano Sivolella** **^2^, Eriberto Bressan** **^2^** **and Barbara Zavan** ^1,*^

^1^ Department of Histology, Microbiology and Medical Biotechnology, University of Padova, Via G. Colombo 3, 35100 Padova, Italy; E-Mails: chiara.gardin@unipd.it (C.G.); letizia.ferroni@unipd.it (L.F.)

^2^ Department of Periodontology, School of Dentistry, University of Padova, Via Venezia 90, 35100 Padova, Italy; E-Mails: lorenzo.favero@unipd.it (L.F.); edoardo.stellini@unipd.it (E.S.); dstomaci@yahoo.it (D.S.); stefano.sivolella@libero.it (S.S.); eriberto.bressan@unipd.it (E.B.)

We apologize for any inconvenience brought to the readers.
